# Gene Regulatory Network Rewiring in the Immune Cells Associated with Cancer

**DOI:** 10.3390/genes8110308

**Published:** 2017-11-07

**Authors:** Pengyong Han, Chandrasekhar Gopalakrishnan, Haiquan Yu, Edwin Wang

**Affiliations:** 1 The State Key Laboratory of Reproductive Regulation and Breeding of Grassland Livestock, Inner Mongolia University, Hohhot 010070, China; hanpengyong@gmail.com; 2 Cumming School of Medicine, University of Calgary, Calgary, AB T2N 1N4, Canada; chandrasekhar.gopala@ucalgary.ca; 3 College of Life Science, Tianjin Normal University, Tianjin 300384, China; 4 Department of Medicine, McGill University, Montreal, QC H3A 0G4, Canada

**Keywords:** tumor infiltrated immune cells, regulatory network, network reprogramming

## Abstract

The gene regulatory networks (GRNs) of immune cells not only indicate cell identity but also reveal the dynamic changes of immune cells when comparing their GRNs. Cancer immunotherapy has advanced in the past few years. Immune-checkpoint blockades (i.e., blocking PD-1, PD-L1, or CTLA-4) have shown durable clinical effects on some patients with various advanced cancers. However, major gaps in our knowledge of immunotherapy have been recognized. To fill these gaps, we conducted a systematic analysis of the GRNs of key immune cell subsets (i.e., B cell, CD4, CD8, CD8 naïve, CD8 Effector memory, CD8 Central Memory, regulatory T, Thelper1, Thelper2, Thelp17, and NK (Nature killer) and DC (Dendritic cell) cells associated with cancer immunologic therapies. We showed that most of the GRNs of these cells in blood share key important hub regulators, but their subnetworks for controlling cell type-specific receptors are different, suggesting that transformation between these immune cell subsets could be fast so that they can rapidly respond to environmental cues. To understand how cancer cells send molecular signals to immune cells to make them more cancer-cell friendly, we compared the GRNs of the tumor-infiltrating immune T cells and their corresponding immune cells in blood. We showed that the network size of the tumor-infiltrating immune T cells’ GRNs was reduced when compared to the GRNs of their corresponding immune cells in blood. These results suggest that the shutting down certain cellular activities of the immune cells by cancer cells is one of the key molecular mechanisms for helping cancer cells to escape the defense of the host immune system. These results highlight the possibility of genetic engineering of T cells for turning on the identified subnetworks that have been shut down by cancer cells to combat tumors.

## 1. Introduction

The human body has an intricate ability to combat genetic disorders such as cancer. The immune system could combat cancer cells by recognizing cancer cells and killing them via sophisticated molecular mechanisms [1]. The immune system has different types of immune cells, which have different functions [2,3]. For example, T cells play an active role in combating cancer cells, while B cells play an innate role in attacking cancer cells. The relationship between immune cells and tumors is evolving with time and cancer progression. Cancer cells bypass immunological suppression and proliferate uncontrollably [4]. In this process, cancer cells may send out molecular signals that can modify the gene regulatory networks of the major immune cells. Interestingly, this process could also provide opportunities for us to identify key molecules to block the immune-escaping processes; for example, by doing so, cancer immunologic therapies have been advanced in the past few years. Immune-checkpoint blockade (i.e., blocking PD-1, PD-L1, or CTLA-4) has shown durable clinical effects in some (but not all) patients with various advanced cancers [5]. At present, one of the critical unanswered challenges in tumor immunity is: What are the key cellular populations, their activation profiles, regulatory mechanisms, and molecular pathways contributing to antagonizing effective anti-tumor immunity? So far, it has been shown that B cells, CD4, CD8 T cells, Dendritic cells (DCs), and regulatory T cells are actively involved in immunotherapy [6]. However, it is not clear how gene regulatory networks (GRN) and molecular pathways are changed in tumor microenvironments (i.e., tumor infiltrating-immune cells (TICs)) and the hosts (i.e., patients).

Thus far, such a systematic analysis of the GRNs in TICs and the hosts (i.e., cancer patients) has not been conducted. Characterizing the GRNs of the immune cell subsets enables us to reveal cell states (i.e., current activity or responding to tissue cues) in different cellular conditions for a given immune cell lineage. The GRNs of the cells can be revealed through chromatin profiling assays. In the past few years, chromatin profiling has become an important tool for studying immune-regulatory networks and chromatin dynamics. Among these, Single-cell Assays of Transposon-Accessible Chromatin using Sequencing (scATAC-seq) have emerged as an effective tool in identifying which regulatory elements are accessible at a particular time at the single-cell level. A single-cell level epigenetic profile could be also obtained using the scATAC-seq approach. Some epigenetic factors have been shown to be key drivers for cancer evolution and immune cells’ changes; thus an understanding of epigenetic variations at the single-cell level could establish the relationship between the enhancers, repressors, modifiers, and their regulated genes and identify potential epigenetic drivers at the single-cell resolution for cancer and immune cells. Further, scATAC-seq requires as little as a few thousand cells (i.e., 1000 to 5000 cells). Thus, scATAC-seq is attractive for sensitive regulatory profile analysis and is ideally suitable for the characterization of rare and interesting immune cell populations [7,8,9]. DNase-seq is another method to identify the location of regulatory regions, although it requires more cells [10].

Network analysis has proved an efficient method to model biological systems [11,12,13,14]. In a GRN, the nodes or vertices of a molecular network represent biomolecules (genes or proteins), while the links represent regulatory relationships. Analysis of GRNs could identify regulatory structures between genes, key regulators, regional subnetworks for certain biological processes, cancer hallmark subnetworks, and network dynamics for network rewiring and network motifs [15]. All of these results could reveal molecular mechanisms, signaling pathways that are associated with cancer hallmarks and cancer patient outcomes [16,17,18,19].

In this study, we first constructed the GRNs for the key innate and adaptive immune cell subsets in peripheral blood mononuclear cells (PBMC) and tumor microenvironments. Then we compared the networks of the immune cells in PBMC by performing network analysis, and, finally, we compared the networks of T cells in PMBC and TICs. We unraveled that the GRNs of the immune cells in the PBMC share key important hub regulators, but their subnetworks for controlling cell type-specific receptors are relatively unique. Further, we showed that cancer cells escape the defense of the host immune system by shutting down certain cellular activities (regions of the GRNs) of the immune cells.

## 2. Materials and Methods

### 2.1. Data Collection

The DNase-seq peak files of human immune cells B, CD4, CD8, DC, NK, regulatory T, Thepler1, Thelper2, and Thelper17 from the PBMC were obtained from ENCODE (Encyclopedia of DNA Elements) [20]. ENCODE contains an extensive collection of human genome regulatory elements that can be utilized for various research purposes. Furthermore, we obtained a portion of peak files from Gene Expression Omnibus (GEO), which is archived to store research-oriented data. The ATAC-seq data used in the present study (GSE89308) is from three melanoma patients (i.e., for PD^hi^ and CD8 T tumor infiltrating cells) and from healthy donors (i.e., for CD8 naïve, CD8 Effector memory, and CD8 Central Memory T cells) [21]. The data source above has been already filtered to have the standard peaks through the systematic and conventional protocol. These high-quality peaks were used for constructing GRNs. 

In order to construct the receptors’ regulatory subnetworks of each immune cell subset, we obtained a list of receptors (i.e., an immune cell receptor list) that are regulated in the immune cells from a recent study [3]. Furthermore, we used UpSet [22] to visualize intersecting sets in order to effectively analyze the unique and common genes, TFs (Transcription factors), and receptors across nine immune cell subsets. This tool could accurately establish Venn diagram relationships across multiple datasets and systematically illustrate the connections between multiple datasets.

Finally, in order to incorporate gene expression profiles into GRNs derived from chromatin profiles, we obtained RNA-seq data from Tirosh et al. (GSE72056) [23] and Pulko et al. (GSE80306) [24]. Gene expression profiles of the T cells of healthy people and the tumor infiltrating T cells have been generated in these studies. Further, we normalized the RNA-seq data and used the seaborn package, which was coded in python to produce a heatmap of the RNA-seq data from above [25].

### 2.2. Regulatory Network Construction

Using the high-quality peaks from either the DNase-seq or the ATAC-seq files for immune cell subsets, we constructed the GRNs of the immune cells. This was conducted by: (1) annotating the downstream genes for each peak; (2) finding the transcription factors that can bind to the peaks; and (3) mapping the downstream genes to the binding transcription factors (TFs).

In order to annotate the downstream genes that are associated with each peak, we employed Hypergeometric Optimization of Motif EnRichment (HOMER) [26], which is an efficient tool for discovering up- and down-stream genes associated with a DNase-seq or the ATAC-seq peak. It is a cluster of C++ and Perl programs, which can be used in command line operating systems such as Linux. We set the cutoff at 2 kb ± of the peak position to filter the genes that are closely associated with a peak. Next, we applied the Multiple Em for Motif Elicitation (MEME) suit to obtain the list of TFs that putatively bind to the peaks [27]. MEME is an efficient tool for uncovering DNA-binding motifs in a class of related protein or DNA sequences. We specifically used the Find Individual Motif Occurrences (FIMO) program of the MEME suite to estimate the occurrence of particular DNA-binding sites and the corresponding proteins that bind to them [28]. FIMO compares the sequences of the peak with the DNA-binding motif databases to establish protein binding regions. The DNA-binding motif database is from the MEME website (http://meme-suite.org/db/motifs/). The TFs were filtered based on the P-value, and a default P-value of 1 × 10^−4^ was taken as the threshold value. 

Once the TFs and their regulated downstream genes have been determined, a shell script was used to link the TFs with their regulated genes; then the GRNs were constructed. Subsequently, to find the subnetworks that are associated with the cell receptors of an immune sunset, we mapped the network genes to the immune cell receptor list and identified their upstream TFs. The unique cell receptors of an immune cell subset and their regulators form a subnetwork of the cell receptors of that immune cell subset.

### 2.3. Analyzing of the TFsand Hub Regulators in the Networks

Once the networks have been constructed, we evaluated the most important TFs by using the network approach. We utilized network analysis algorithms to find out which TF had the most downstream genes under its control for each immune cell subset.

Further, to compare the results obtained from the analysis mentioned above, we employed a ‘PageRank algorithm’ [29] to identify hub regulators.

### 2.4. Network Visualization and Comparison

To conduct an analysis and comparison of the GRNs and regulatory circuits, we applied Gephi [30] and Cytoscape [31] the GRNs for analyses. Both tools are effective for analyzing and visualizing interactive networks and biological pathways. Moreover, they were used for integrating and annotating networks with various biological data such as gene expression profiles. The GRNs of each immune cell subset were analyzed using a shell script and then visualized in Gephi. Furthermore, Cytoscape was used to identify key regulators of the GRNs.

To elucidate the signaling pathways that are associated with uniquely regulated genes in the GRN of the T cells in PBMC (peripheral blood mononuclear cell) versus the TIC T cells, we conducted an analysis of their GRNs using Enrichr [32]. Enrichr is an extensive web server to perform gene set enrichment analyses with over 184 annotated gene sets from 102 gene set libraries [33].

## 3. Results 

### 3.1. Characterize Gene Regulatory Networks of Key Immune Cells Associated with Cancer Immunotherapy 

**Figure 1:**
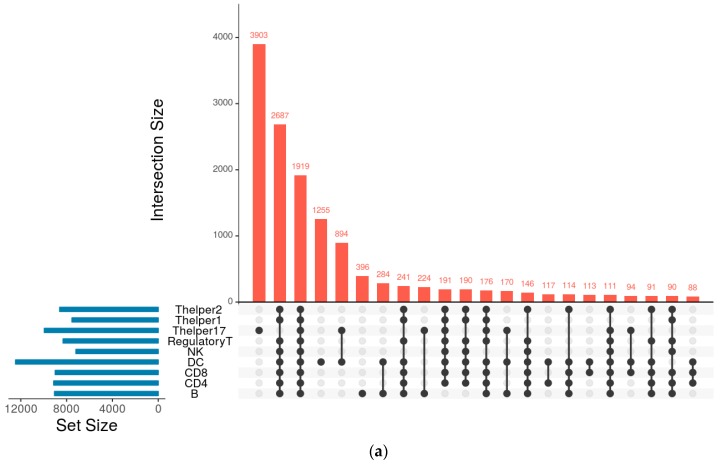

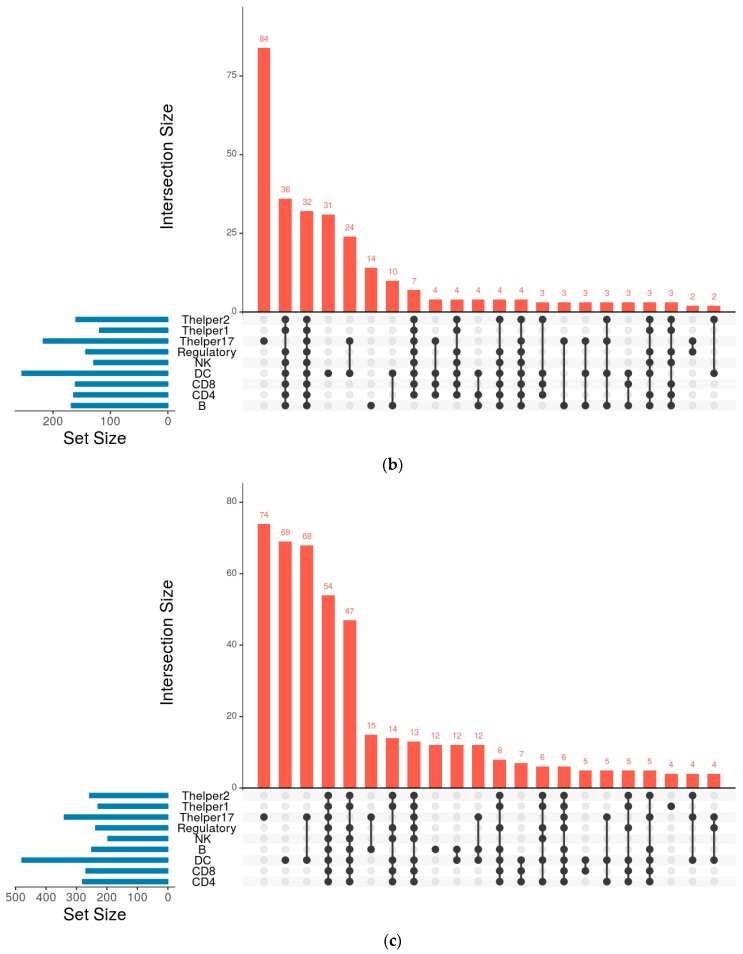
Unique and overlapping genes, receptors, and transcription factors (TFs) between immune cells. The figure reveals the number of genes that are unique to each cell subset or common across different cell types/subsets. The red histogram represents the size of the elements that are present in the relation, which is depicted as the black dots between the cell types (it is of note that elements with low numbers have not been depicted), while the blue bar graph specifies the total number of elements that are present in each set. (**a**) The intersections and unique genes for all the cell types; (**b**) The variations of transcription factors across all the immune cell types; (**c**) The common and unique receptors across all the immune cell types.

The key immune cells associated with cancer immunotherapy have been reported. Among them, we selected nine key immune cell subsets in PBMC (Table 1), the chromatin profiles of which have been determined by either DNase-seq or ATAC-seq. To construct the GRN of an immune cell subset, we obtained the DNase-seq or ATAC-seq peak files of the immune cell subset to identify their chromatin open regions. Based on these regions, we scanned the DNA-binding motifs and their associated TFs (for details, see Methods), and then constructed the GRN. The networks of the nine immune cell subsets contain 8000 to 12,000 nodes (genes). However, they are not quite similar. Each of the immune cells has its own unique genes and genes that are common across the immune cell subsets. For instance, Thelper17 has around 3900 unique genes, while NK cell has only 39 unique genes in the GRNs. The characteristics of the key immune cell subsets’ GRNs have been summarized in Table 1 and Figure 1a. Table 1 contains the genes annotated for each immune cell subset and the list of the genes that are unique to each immune cell subset. Furthermore, in order to find out the biological pathways that are associated with the uniquely regulated genes of the immune cells, we conducted enrichment analyses using Enchr on the unique genes of each immune cell’s GRN. Table 2 listed the most enriched signaling pathways for each immune cell subset. For example, both the IL-2 receptor beta chain for T cell activation pathway and the PDGF signaling pathway are enriched in B cells. The Ras pathway, the ErbB1 downstream signaling pathway, and the mTOR signaling pathway are enriched in CD4 T cells. In the NK and regulatory T cells, the ATR (ataxia telangiectasia and Rad3-related protein) signaling pathway is enriched. These results suggest that the immune cell subsets have specific signaling pathways for cell-cell communications.

TFs play a prominent role in controlling the functionality of cells. GRNs encode the key global regulators and the hierarchy structure in the networks of the immune cells [34]. To find out the most important TFs for each immune cell subset, we analyzed the GRN of each immune cell subset and found the key regulators that control the networks. Table 1 illustrates the total number of TFs that can be found across each immune cell subset and the TFs that are unique to each of them. The distribution of TFs across the cell subsets is illustrated in Figure 1b. Further, we employed the network analysis algorithm to find the top 20 important TFs that influence the most downstream genes. Table 3 showed the top 20 TFs (i.e., based on their regulated gene numbers) in each immune cell subset. The TFs *ELF2*, *KLF14*, *ZFX*, *ZN639*, *PLAG1*, *TBX1*, *KLF6*, *ZN148*, *KLF4*, *EGR4*, *TBX15*, *WT1*, *KLF16*, *THAP1*, *SP3*, *SP4*, *MAZ*, *SP2*, *TFDP1*, *EGR1*, and *SP1* are shared by immune cell subsets such as B, CD4, CD8, DC, NK, Regulatory T, Thelper1, and Thelper2 cells. However, Thelper17 has unique TFs such as *PROX1*, *MNT*, *PURA*, and *PAX5*. To substantiate the results obtained from above analysis, we used another computational procedure, the ‘PageRank’ algorithm [29]. Table 3 lists the top 20 TFs obtained from both methodologies. The results (Table 3) showed that the TFs derived from the two methods are quite indistinguishable.

**Figure 2:**
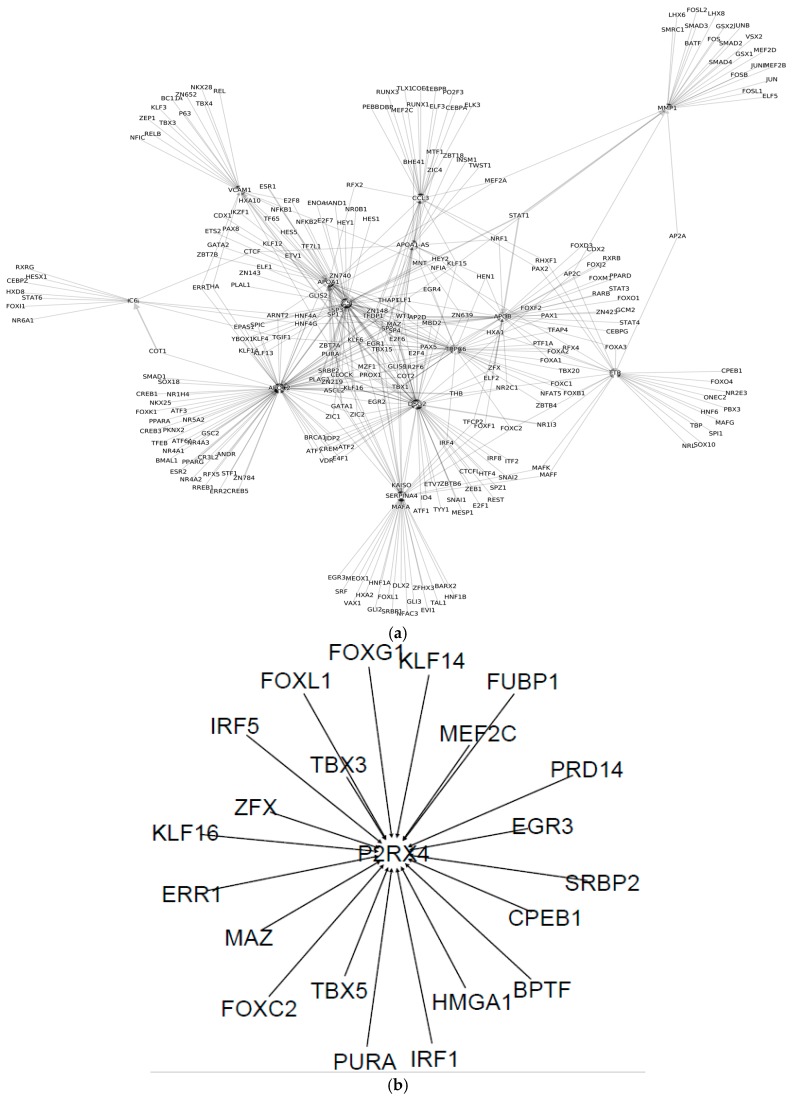

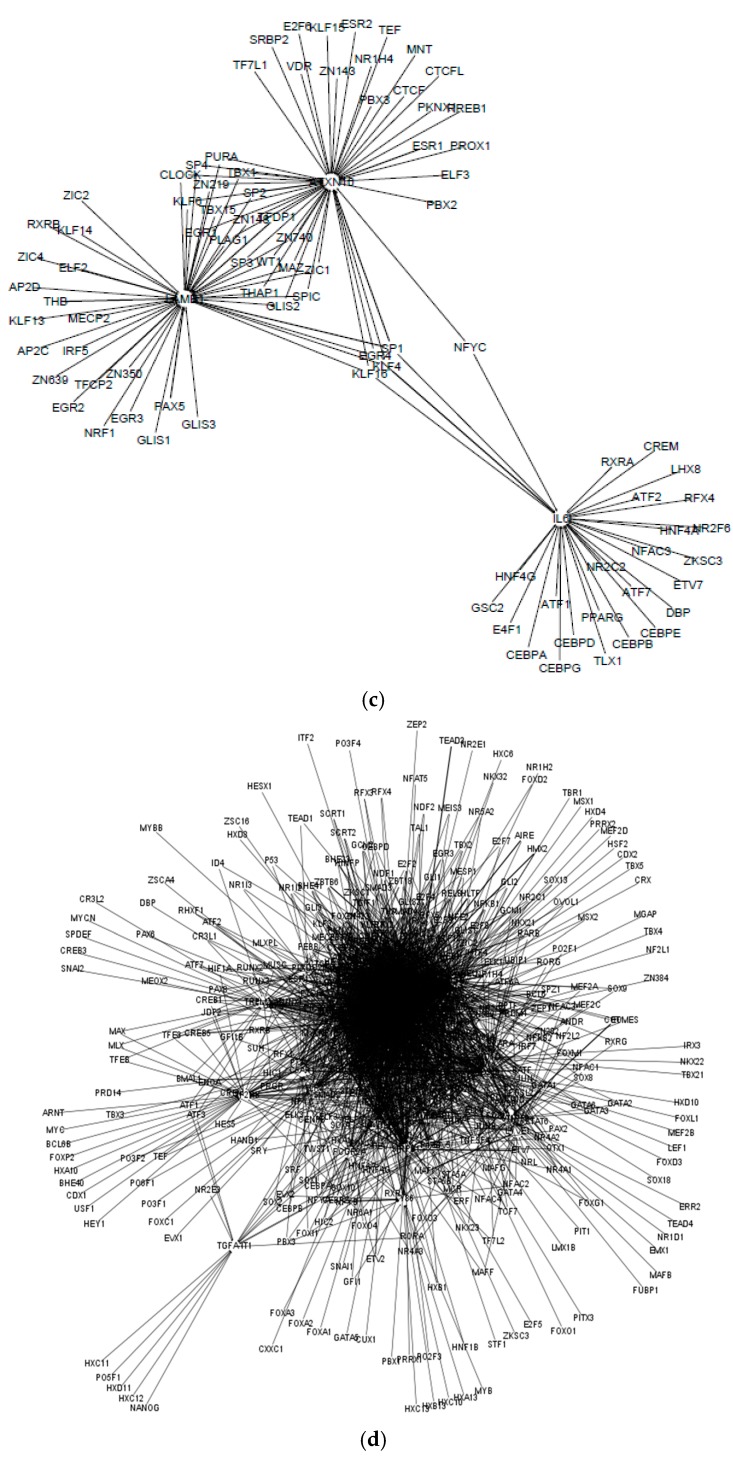

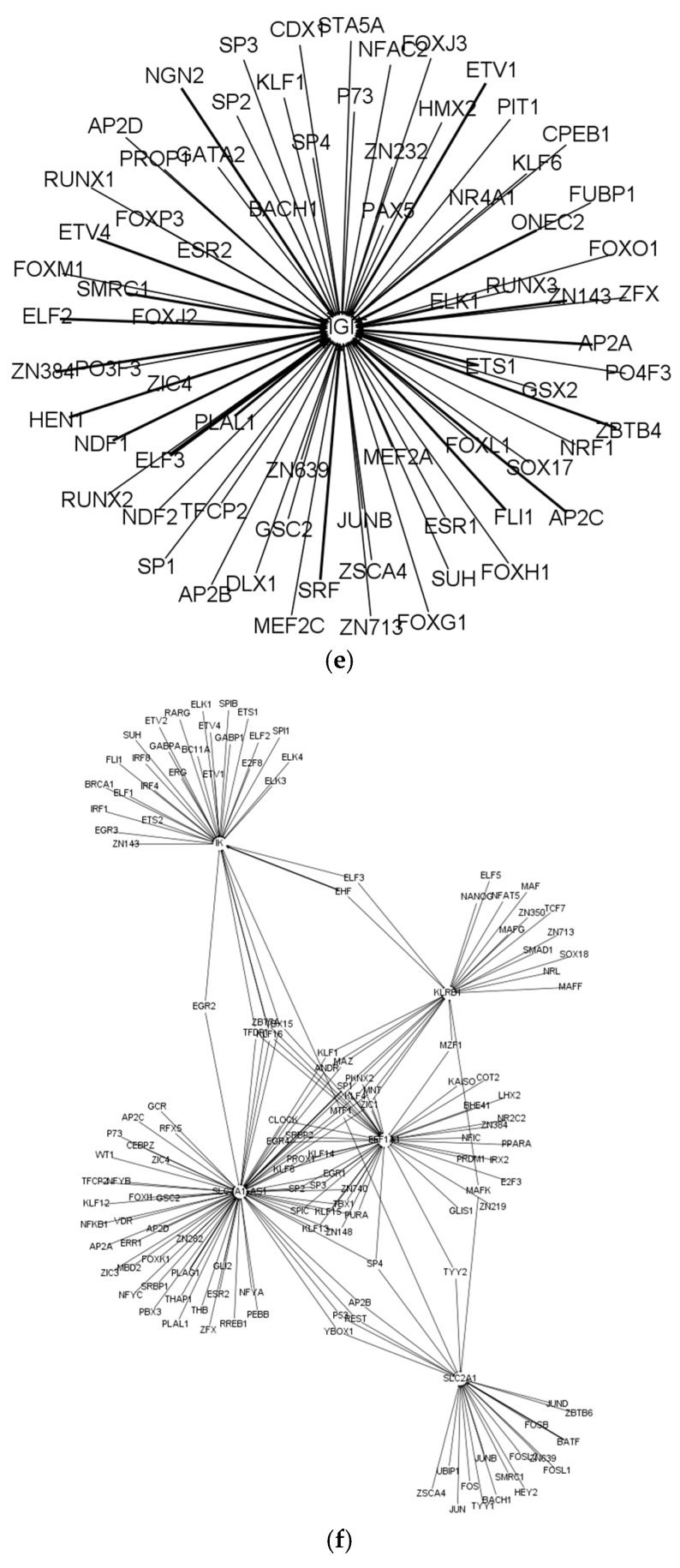

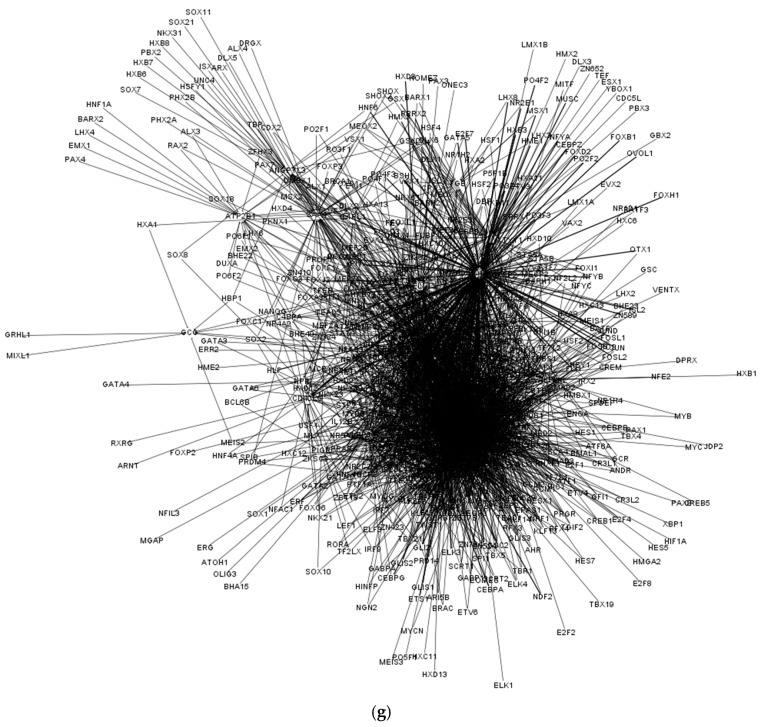
Receptor regulatory subnetworks of normal immune cells. In the network, nodes represent genes and transcription factors (TFs), while links represent gene regulatory relations. Arrows represent TFs regulating genes. The receptor regulatory subnetworks of: (**a**) B cell; (**b**) CD4 cell; (**c**) CD8 cell; (**d**) DC cell; (**e**) regulatory T cell; (**f**) T-helper1 cell; and (**g**) T-helper17 cell. Subnetworks have not been constructed for the immune cell subsets that do not have specific cell receptors.

To examine the cell receptors of the immune cells, which, not only represent cell-cell communication, but also identify the GRNs of the immune cell subsets, we analyzed the receptor subnetwork for each immune cell subset. The results are shown in Table 1 and Figure 1c. The receptor subnetworks of the immune cell subsets are very different (Figure 2). For example, although DC and Thelper17 cells have 68 receptors, respectively, none of them are shared by the two immune cell subsets. On the other hand, Thelper2 and NK cells do not have any unique receptors. Interestingly, we found that the P2RX4 receptor is specific to CD4 cells, ESX1 is specific to regulatory T cells, and the IL6, ATXN10, and LAMB1 receptors are specific to CD8 cells. These specific receptors and their associated subnetworks could determine their cell identity or cellular state.

In summary, these results showed that most of the key regulators are similar in the immune cell GRNs, suggesting that the top regulators have been shared by many immune cell subsets. However, the regulated genes of the key TFs change a lot. Further, the signaling pathways that are associated with uniquely regulated genes for each immune cell subset are quite different, which means that the key regulators may control multiple different pathways. However, the specific cell receptors and the subnetworks of the immune cell subsets could operate in different immune cells to determine their identity.

### 3.2. Melanoma Cells Shut Down Many Network Activities of the CD8 T Cells 

The CD8 T cell is one of the major immune cells that play a key role in killing cancer cells. However, it has been quite elusive how the cancer cell has evaded the immune system. To understand the underlying molecular mechanisms, we constructed and compared the GRNs for PD^hi^ CD8 T tumor infiltrating cells (i.e., CD8 T cells with a high expression of PD-1 in melanoma) and three corresponding T cell subsets (i.e., CD8 naïve, CD8 Effector memory, and CD8 Central Memory T cells) in the PBMC of healthy people. Interestingly, we found that the GRN network size of the PD^hi^ CD8 T cells changed compared to the GRNs of each PBMC’s T cell subset. For example, the GRNs of the CD8 Central Memory and CD8 Effector memory have three million gene regulatory pairs, whereas the GRN of the PD^hi^ CD8 T cells has 2.8 million gene regulatory pairs. These changes suggest that cancer cells could shut down specific T cell activities (i.e., regional subnetworks), which could be responsible for attacking cancer cells during tumorigenesis. 

To carefully examine the differences in the GRNs of the PD^hi^ CD8 T cells and the three corresponding T cell subsets of the healthy people, we focused on comparing their cell receptors and their associated subnetworks. To do so, we mapped the immune cell receptor list to each GRN and constructed cell receptor subnetworks (see Methods), and we found that specific receptors and their subnetworks are different between the PD^hi^ CD8 T cell and its corresponding PBMC T cells.

**Figure 3:**
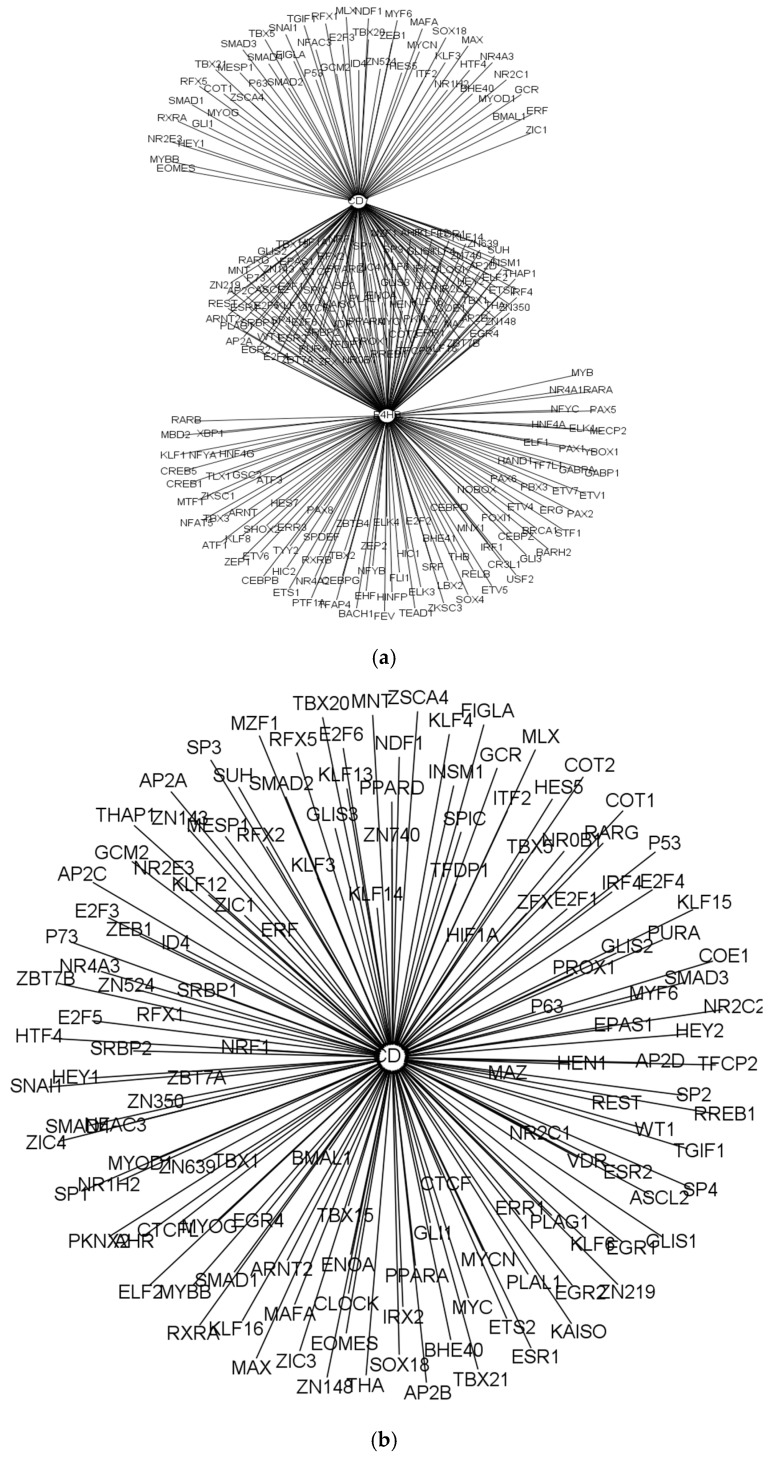

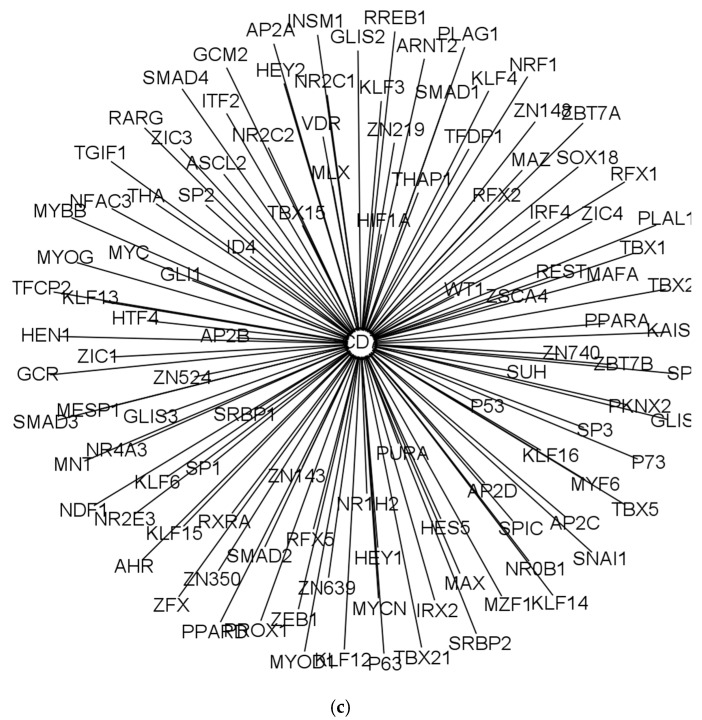
Deferential receptor regulatory subnetworks. In the network, nodes represent genes and transcription factors (TFs), while links represent gene regulatory relations. Arrows represent TFs regulating genes. The differential receptor regulatory network derived from the GRN of the PD^hi^ CD8 T cells with the GRN of the: (**a**) CD8 naïve cells; (**b**) CD8 effector memory cells; and (**c**) CD8 central memory T cells.

**Figure 4:**
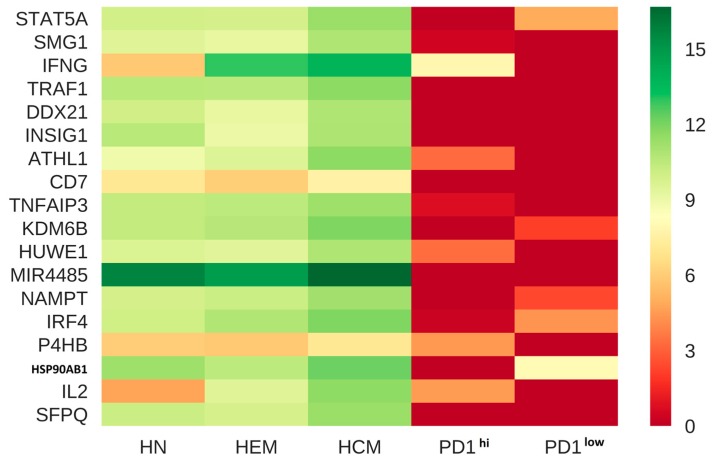
A heatmap generated using significantly modulated genes (*p* < 0.01) across the T cells of healthy people and tumor infiltrating CD8 T cells. HN, HEM and HCM represent human Naïve T cell, human effector T cell, and human memory T cell, respectively from healthy people, while PD1^hi^ and PD1^lo^ represent the tumor-infiltrating CD8 T cells with high- and low-expression of PD1, respectively. The rows are modulated genes, and colors represent the gene expression levels. The darker shade of red indicates a low-expressed pattern, while a green shade depicts a high-expressed pattern.

Figure 3 showed the cell receptor subnetworks, which are differential receptor networks between the PD^hi^ CD8 T cells and each of the corresponding PBMC T cells. Figure 3a illustrated the differential receptor regulatory network derived from the GRNs of the CD8 naïve cells and the PD^hi^ CD8 T cells and showed that both receptors, P4HB and CD7, which are originally regulated in the 179 and 332 TFs, respectively, in the GRN of the CD8 naïve cells, disappeared in the GRN of the PD^hi^ CD8 T cells. We found that the receptor CD7, which is activated and regulated by 493 and 488 TFs in the differential receptor regulatory networks derived from the GRNs of the CD8 effector memory cells and the PD^hi^ CD8 T cells (Figure 3b) and from the GRNs of the CD8 central memory T cells and the PD^hi^ CD8 T cells (Figure 3c), respectively, was inactivated in the GRN of the PD^hi^ CD8 T cells. These results suggest that both P4HB and CD7 receptors could be associated with the process of tumor infiltration for reducing the immunological function of the T cells. Moreover, RNA-Seq data from Tirosh et al. and Pulko et al. clearly showed that CD7 and P4HB have higher expression levels in the T cells of the healthy people but lower expression levels in the tumor-infiltrating T cells (Figure 4). This result validated the findings from our GRN analysis, mentioned above. Furthermore, an enrichment analysis of the differentially modulated genes between the T cells (Table 4) revealed that interleukin signaling pathways are highly enriched in the tumor-infiltrating T cells.

## 4. Discussion

Cancer immunologic therapies have been advanced in the past few years. Immune-checkpoint blockade (i.e., blocking PD-1, PD-L1, or CTLA-4) has shown durable clinical effects in some patients with various advanced cancers. Although amazing clinical responses have been observed with these therapies, the fact remains that only a relatively small subset of patients derives substantive clinical benefit from the therapy. There are major gaps in our knowledge of immunotherapy. One of the critical unanswered challenges is how immune cells become cancer-cell friendly and do not attack cancer cells. To uncover the underlying molecular mechanisms, we constructed and analyzed the GRNs of the key immune cell subsets associated with cancer immunologic therapies. We first analyzed the GRNs of the key PBMC’s immune cell subsets, including B cell, CD4, CD8, CD8 naïve, CD8 Effector memory, CD8 Central Memory, regulatory T, Thelper1, Thelper2, Thelp17, and NK and DC cells to understand their activation profiles, regulatory mechanisms, and molecular pathways. It should be noted that this is the first study to systematical analyze the GRNs of immune cells.

We constructed GRNs using ATAC-seq and DNase-seq data. To check that the inferred regulatory relationships are functional, it is essential to examine the GRNs using gene expression profiles. Previously, we had used the ATAC-seq, DNase-seq, and gene expression data of the immune cells from the ENCODE database to check the parameters of the MEME tool for filtering TF-binding motifs. For example, by changing the parameters, we were looking for a GRN in which most of the both predicted TFs and their regulated genes are expressed (via checking the gene expression profiles of the same cell for that GRN) and most of the non-predicted TFs and their potentially regulated genes are not expressed (unpublished results). In this study, we applied the appropriate parameters (i.e., we used the *p*-value, 1 × 10^−4^, for filtering TF-binding sites) to construct the GRNs.

By comparative-network analysis of the GRNs of the key immune cells, we showed that most of the GRNs share key important hub regulators and that the differences between the immune cells are from the local regulatory subnetworks, which control the cell type-specific cell receptors. The cell receptors are the hallmark of each immune cell subset and allow us to pinpoint the immune cell subsets. In fact, these cell receptors could be used as cell markers to study immune cell subpopulations. The network structures and organizations of the immune cells where global regulators are shared in the GRNs and the subnetworks of the local, cell type-specific cell receptors have specificity, which suggests that the transformation of the immune cell subsets or cell types is relatively easily: the key hub regulators can be kept and the local regulatory networks can be changed between immune cell subsets. This nature of the GRNs of the immune cell subsets suggests that immune cell subsets have plasticity and could have a fast molecular mechanism for cell transformation between the immune cell subsets. This fast cell transformation mechanism allows rapid responses to distinct environmental signal cues.

To understand how cancer cells educate immune cells to make them more cancer-cell friendly, we constructed the GRNs of the TICs and compared their corresponding immune cells in PBMC in the same melanoma patient. In human melanoma, we found that the GRN of the tumor-infiltrating T cells has reduced its network size compared to those of the corresponding T cell subsets in PBMC. These results clearly showed that cancer cells send signals to shut down many cellular activities of the TIC T cells. Based on this result, we highlighted that one of the key underlying molecular mechanism for making T cells more cancer-cell friendly is shutting down a lot of a T cell’s cellular activities, which could originally allow T cells to recognize and attack cancer cells during tumor infiltrating T cells. Carefully analyzing of the shutdown-subnetwork of the TIC’s T cells suggests that CD7 and P4HB receptor subnetworks have been turned off by cancer cells.

Galectin-1 (LGALS1) is selectively upregulated in Treg cells [35]. LGALS1 binds to glycoproteins on the T cell surface receptor CD7. It has been shown that upregulated CD7 in T cells can trigger a pro-apoptotic signal during LGALS1-induced T cell death. P4HB is a hormone binding protein that can bind to Galectin-9 [36]. Both Galetin-9 and LGALS1 can trigger T cell death. It has been shown that the exhausted T cells express high levels of PD-1 by losing CD7 and P4HB expression. The will decrease the level to trigger T cell death, which may make the T cells get into a proliferative defect, exhausted state. Tumor-infiltrating T cells have two fractions: PD1^hi^ (T cells with high-expressed PD1) and PD1^lo^ T cells (T cells with low-expressed PD1) (see Ref 23). An enrichment analysis of them using RNA-seq data (Table 4) showed that both the glucocorticoid receptor regulatory network and the ATF-2 transcription factor network are enriched in the PD1^hi^ T cells. The pathways of IL12-mediated signaling, IL2 signaling events mediated by STAT5, and calcineurin-regulated NFAT-dependent transcription are enriched in most of the functional CD8 T cells (functional CD8 vs exhausted CD8 T cells). Philip found that a cell subset of PD1^hi^ T cells had higher levels of CD38 and CD101 and lower levels of CD5, which could be cell markers for the exhausted T cells [23]. Pauken et al. found that PD1 blockade can reprogram the epigenetic landscapes of exhausted T cells to functionally effect T cells at some level [37]. These results could suggest that losing CD7 and P4HB expression in the PD1^hi^ T cells could induce the exhausted state of the T cells.

In addition, CD7 is specifically expressed in T and NK cells. Its expression could activate T cells. Further, studies [38] have shown that a ligand for CD7, SECTM1 (secreted and transmembrane protein 1), is highly expressed in many tumors, including melanoma cells. The interaction of the SECTM1 and CD7 significantly increases monocyte migration by activation of the PI3K pathway. Thus, we suspect that tumor cells could send a certain signal to turn off or down-regulate CD7 expression in the TIC T cells so that it could reduce the migration of the T cells into the tumors. We envisioned that if we could genetically engineer the T cells so that we can constitutively turn on the subnetworks that have been shut down by cancer cells, we could let T cells recognize and attack cancer cells and pave a new way to treat tumors using T cell genetic engineering.

## Figures and Tables

**Table 1:** The numbers of genes, uniquely regulated genes, cell receptors, and transcription factors in the gene regulatory networks of immune cells.

Cell Type	Numbers of Annotated Genes	Number of Genes that Are Unique to Each Cell Type	Numbers of Receptors	Numbers of Receptor that Are Unique to Each Cell Type	Numbers of TFs	Numbers of TFs that Are Unique to Each Cell Type
B	9058	396	250	12	168	14
CD4	9128	68	280	1	164	2
CD8	8997	71	269	3	161	1
DC	12,459	1255	480	69	254	34
NK	7186	39	197	0	129	1
Regulatory T	8309	72	238	1	143	1
Thelper1	7533	41	229	4	119	1
Thelper2	8606	36	257	0	160	1
Thelper17	9947	3903	341	74	217	84

**Table 2:** Enriched specific signaling pathways in the gene regulatory networks of immune cells.

**B-Cell**	
**Pathway Name**	***p*-Value**
TPO Signaling Pathway	1.95 × 10^−4^
IL-2 Receptor Beta Chain in T Cell Activation	4.45 × 10^−^^5^
PDGF Signaling Pathway	1.16 × 10^−3^
Role of Calcineurin-dependent NFAT (Nuclear factor of activated T-cells) signaling in lymphocytes	1.23792 × 10^−4^
Phosphoinositides and their downstream targets	5.69 × 10^−4^
**CD4**	
**Pathway Name**	***p*-Value**
ErbB1 downstream signaling	3.10 × 10^−10^
mTOR signaling pathway	6.80 × 10^−9^
Ras Pathway	6.80 × 10^−9^
IL2-mediated signaling events	3.17 × 10^−8^
PDGFR (Platelet-derived growth factor receptors)-beta signaling pathway	1.13 × 10^−7^
**CD8**	
**Pathway Name**	***p*-Value**
FoxO family signaling	1.25 × 10^−8^
Fanconi anemia pathway	3.60 × 10^−6^
E2F transcription factor network	4.26 × 10^−6^
**Dendritic Cell**	
**Pathway Name**	***p*-Value**
BCR signaling pathway	0.81 × 10^−8^
CCKR signaling map	6.83 × 10^−7^
TCR signaling in naive CD4+ T cells	1.455 × 10^−6^
CXCR4-mediated signaling events	1.66 × 10^−6^
FoxO family signaling	4.05 × 10^−6^
Class I PI3K signaling events	4.05 × 10^−6^
**NK cells**	
**Pathway Name**	***p*-Value**
ATR signaling pathway	3.25 × 10^−8^
FoxO family signaling	1.97 × 10^−7^
CCKR (cholecystokinin receptor) signaling map signal transduction	4.29 × 10^−7^
**Regulatory T cell**	
**Pathway Name**	***p*-Value**
ATR signaling pathway	6.72 × 10^−7^

**Table 3:** Key regulators in the gene regulatory networks of immune cells.

Cell Type	Computational Method Employed	Key_Regulators
B	Network-analysis	*SP1, EGR1, TFDP1, SP2, SP4, MAZ, SP3, THAP1, KLF16, TBX15, WT1, KLF4, ZN148, EGR4, TBX1, PLAG1, KLF6, ZN639, ZFX, KLF14*
	PageRank algorithm	*SP1, EGR1, TFDP1, SP4, SP2, MAZ, SP3, THAP1, KLF16, WT1, TBX15, KLF4, ZN148, EGR4, TBX1, ZFX, KLF6, ZN639, ELF2, PLAG1*
CD4	Network-analysis	*SP1, EGR1, TFDP1, SP2, MAZ, SP4, SP3, THAP1, KLF16, TBX15, WT1, EGR4, KLF4, ZN148, TBX1, KLF6, ZN639, PLAG1, ZFX, ELF2,*
	PageRank algorithm	*SP1, EGR1, TFDP1, MAZ, SP2, SP4, SP3, THAP1, KLF16, TBX15, WT1, EGR4, KLF4, ZN148, TBX1, KLF6ZN639, ZFX, PLAG1, ELF2*
CD8	Network-analysis	*SP1, EGR1, TFDP1, SP2, MAZ, SP4, SP3, THAP1, KLF16, WT1, TBX15, EGR4, KLF4, ZN148, KLF6, TBX1, PLAG1, ZN639, ZFX, KLF14*
	PageRank algorithm	*SP1, EGR1, TFDP1, SP2, SP4, MAZ, SP3, THAP1, KLF16, WT1, TBX15, EGR4, KLF4, ZN148, KLF6, TBX1, ZN639, PLAG1, ZFX, ELF2*
DC	Network-analysis	*SP1, EGR1, TFDP1, SP2, SP4, MAZ, SP3, THAP1, KLF16, TBX15, WT1, EGR4, ZN148, TBX1, KLF6, PLAG1, KLF4, ZFX, ZN639, AP2D*
	PageRank algorithm	*SP1, EGR1, TFDP1, SP2, SP4, MAZ, SP3, THAP1, KLF16, TBX15, WT1, EGR4, TBX1, ZN148, KLF6, KLF4, PLAG1, ZN639, ZFX, ELF2*
NK	Network-analysis	*SP1, EGR1, TFDP1, SP2, MAZ, SP4, SP3, THAP1, KLF16, TBX15, WT1, KLF4, EGR4, ZN148, TBX1, KLF6, ELF2, ZN639, PLAG1, ZFX*
	PageRank algorithm	*SP1, EGR1, TFDP1, MAZ, SP2, SP4, SP3, THAP1, KLF16, TBX15, WT1, KLF4, EGR4, TBX1, ZN639, ZN148, KLF6, ELF2, ZFX, PLAG1*
Regulatory T	Network-analysis	*SP1, EGR1, TFDP1, SP2, MAZ, SP4, SP3, THAP1, KLF16, TBX15, WT1, KLF4, ZN148, EGR4, TBX1, KLF6, PLAG1, ZN639, ELF2, ZFX*
	PageRank algorithm	*SP1, EGR1, TFDP1, SP2, SP4, MAZ, SP3, THAP1, KLF16, TBX15, WT1, KLF4, EGR4, ZN148, TBX1, ZN639, KLF6, ELF2, ZFX, PLAG1*
Thelper17	Network-analysis	*SP1, EGR1, MAZ, SP2, TFDP1, SP4, SP3, TBX15, PLAG1, KLF16, TBX1, PAX5, PURA, ZN148, THAP1, WT1, KLF15, MNT, ZFX, AP2D*
	PageRank algorithm	*SP1, EGR1, MAZ, SP2, TFDP1, SP4, SP3, TBX15, PLAG1, KLF16, TBX1, PAX5, PURA, ZN148, THAP1, MNT, WT1, KLF15, ZFX, AP2D*
Thelper1	Network-analysis	*SP1, EGR1, TFDP1, SP2, MAZ, SP4, SP3, THAP1, KLF16, TBX15, WT1, KLF4, ZN148, TBX1, ELF2, KLF6, EGR4, ZN639, KLF14, ZFX*
	PageRank algorithm	*SP1, EGR1, TFDP1, SP2, MAZ, SP4, SP3, THAP1, KLF16, TBX15, WT1, KLF4, ELF2, ZN148, ZN639, KLF6, EGR4, TBX1, ZFX, KLF14*
Thelper2	Network-analysis	*SP1, EGR1, TFDP1, SP2, SP4, MAZ, SP3, THAP1, KLF16, WT1, TBX15, KLF4, EGR4, ZN148, KLF6, TBX1, ZN639, KLF14, ELF2, PLAG1*
	PageRank algorithm	*SP1, EGR1, TFDP1, SP2, SP4, MAZ, SP3, THAP1, KLF16, WT1, TBX15, KLF4, EGR4, ZN639, KLF6, ZN148, TBX1, ELF2, ZFX, KLF14*

The first column depicts the various normal immune cell types used for our analysis, while the second column highlights the computational methods employed to scrutinize the most important TFs. Finally, the third column enlists the TFs that are found by the corresponding computational methods.

**Table 4:** Enriched specific signaling pathways in the differentially expressed genes between the T cells of healthy people and tumor infiltrating CD8 T cells.

Cell Type	Name	*p*-Value
**HCM vs PD1^lo^**	Calcineurin-regulated NFAT (Nuclear factor of activated T-cells) -dependent transcription in lymphocytes	1.443 × 10^−12^
	IL2 signaling events mediated by STAT5	1.34 × 10^−12^
	Downstream signaling in naive CD8+ T cells	1.036 × 10^−8^
	IL12-mediated signaling events	2.724 × 10^−8^
	FoxO family signaling	3.688 × 10^−8^
**HCM vs PD1^hi^**	Calcineurin-regulated NFAT-dependent transcription in lymphocytes	9.083 × 10^−13^
	IL2 signaling events mediated by STAT5	4.072 × 10^−11^
	GMCSF-mediated signaling events	8.323 × 10^−9^
	IL2-mediated signaling events	2.378 × 10^−8^
	AP-1 transcription factor network	5.012 × 10^−7^
**HEM vs PD1^lo^**	Calcineurin-regulated NFAT-dependent transcription in lymphocytes	6.401 × 10^−16^
	IL2 signaling events mediated by STAT5	1.157 × 10^−12^
	Downstream signaling in naive CD8+ T cells	6.909 × 10^−11^
	IL12-mediated signaling events	4.682 × 10^−10^
	AP-1 transcription factor network	2.142 × 10^−8^
**HEM vs PD1^hi^**	Calcineurin-regulated NFAT-dependent transcription in lymphocytes	2.304 × 10^−14^
	AP-1 transcription factor network	1.869 × 10^−9^
	IL2 signaling events mediated by STAT5	1.363 × 10^−10^
	IL2-mediated signaling events	4.521 × 10^−8^
	IL12-mediated signaling events	1.329 × 10^−7^
**HN vs PD1^lo^**	Validated targets of C-MYC transcriptional activation	5.009 × 10^−7^
	Glucocorticoid receptor regulatory network	5.60 × 10^−5^
	FoxO family signaling	4.64 × 10^−5^
	Role of Calcineurin-dependent NFAT signaling in lymphocytes	9.98 × 10^−5^
	IL12-mediated signaling events	3.25 × 10^−4^
**HN vs PD1^hi^**	Calcineurin-regulated NFAT-dependent transcription in lymphocytes	8.443 × 10^−8^
	AP-1 transcription factor network	3.14 × 10^−6^
	IL2 signaling events	6.686 × 10^−7^
	IL5-mediated signaling events	2.65 × 10^−5^
	IL2-mediated signaling events	4.72 × 10^−5^
**PD1hi vs PD1^lo^**	IL12 signaling mediated by STAT4	5.04 × 10^−4^
	IL12-mediated signaling events	3.60 × 10^−3^
	TCR signaling in naive CD4+ T cells	4.00 × 10^−3^
	Glucocorticoid receptor regulatory network	8.30 × 10^−3^
	ATF-2 transcription factor network	7.50 × 10^−2^

HN, HEM and HCM represent human Naïve T cell, human effector T cell, and human memory T cell, respectively, from healthy people, while PD1^hi^ and PD1^lo^ represent the tumor infiltrating CD8 T cells with high- and low-expression of PD1, respectively.
